# Pomegranate as a Potential Alternative of Pain Management: A Review

**DOI:** 10.3390/plants9040419

**Published:** 2020-03-30

**Authors:** José Antonio Guerrero-Solano, Osmar Antonio Jaramillo-Morales, Claudia Velázquez-González, Minarda De la O-Arciniega, Araceli Castañeda-Ovando, Gabriel Betanzos-Cabrera, Mirandeli Bautista

**Affiliations:** 1 Academic Area of Pharmacy, Institute of Health Sciences, Autonomous University of the State of Hidalgo, Mexico, San Agustin Tlaxiaca, Hidalgo 42160, Mexico; jose_guerrero@uaeh.edu.mx (J.A.G.-S.); claudiav@uaeh.edu.mx (C.V.-G.); mina@uaeh.edu.mx (M.D.l.O.-A.); 2 Academic Area of Food Chemistry, Institute of Basic Sciences and Engineering, Autonomous University of the State of Hidalgo, Mexico, Pachuca- Tulancingo km 4.5 Carboneras, Pachuca de Soto, Hidalgo 42184, Mexico; ovandoa@uaeh.edu.mx; 3 Academic Area of Nutrition, Institute of Health Sciences, Autonomous University of the State of Hidalgo, Mexico, San Agustin Tlaxiaca, Hidalgo 42160, Mexico; gbetanzo@uaeh.edu.mx

**Keywords:** Pomegranate, *Punica granatum* L., pain, antinociceptive

## Abstract

The use of complementary medicine has recently increased in an attempt to find effective alternative therapies that reduce the adverse effects of drugs. *Punica granatum* L. (pomegranate) has been used in traditional medicine for different kinds of pain. This review aims to explore the scientific evidence about the antinociceptive effect of pomegranate. A selection of original scientific articles that accomplished the inclusion criteria was carried out. It was found that different parts of pomegranate showed an antinociceptive effect; this effect can be due mainly by the presence of polyphenols, flavonoids, or fatty acids. It is suggested in the literature that the mechanisms of action may be related to the activation of the L-arginine / NO pathway, members of the TRP superfamily (TRPA1 or TRPV1) and the opioid system. The implications for the field are to know the mechanisms of action by which this effect is generated and thus be able to create alternative treatments for specific types of pain, which help alleviate it and reduce the adverse effects produced by drugs. The results propose that pomegranate and secondary metabolites could be considered in the treatment of inflammatory, nociceptive, and neuropathic pain.

## 1. Introduction

Pomegranate (*Punica granatum* L.), the name derived from the Latin “pomus” and “granum,” whose meaning is apple with grains, is the edible fruit of the pomegranate tree belonging to the Punicaceae family (this genus derives from the Phoenicians, who were active diffusers of their cultivation) [1]. The pomegranate tree is native to North Africa and the Middle East, although its cultivation has been worldwide, and some antecedents date back to ancient cultures (Babylonian, Greek, Hebrew, Persian, and Chinese) [2,3,4]. Besides the input to the human diet of pomegranate fruit, other parts of the tree, like leaves, roots, or flowers have been used in traditional medicine in many cultures because of its beneficial effects on health [5]. The most widespread use in the world of *Punica granatum* fruit is as an antimicrobial agent [6,7]. However, the juice, whole fruit, flowers, and roots have many other uses whose effects are reported in scientific reports: astringent, blisters, milkweed, cough [8], abortive [9], burns [10], hypoglycemic [11,12,13], dyslipidemia [14,15], antihypertensive [16,17], weight reduction [15,18], antiatherosclerotic [19,20], against erectile dysfunction [21] anti-inflammatory [22,23], metabolic syndrome [24,25], insulin resistance [26], anti-teratogenic [27,28,29], nephroprotective [30], antimicrobial, anti-fungic [23,31], wound healing [32], oral health [33] and pain, and effects that will be discussed in detail in this document. Thus, pain is defined by the International Association for the Study of Pain (IASP) as an unpleasant sensory and emotional experience associated with actual or potential tissue damage, or described in terms of such damage [34], the neural process of encoding noxious stimuli is called: nociception [35,36]. Woolf [37] classified pain according to its neurophysiological mechanism as 1) Nociceptive; a transient pain in response to a noxious stimulus, 2) Inflammatory; a spontaneous and hypersensitivity to pain in response to tissue damage and inflammation, 3) Neuropathic; a spontaneous pain and hypersensitivity to pain in association with damage or injury to the nervous system and 4) Functional; hypersensitivity to pain resulting from abnormal central processing. Despite the existence of a wide variety of therapeutic options used for the treatment of pain, this continues to be the main cause of medical consultation and is on the list of priority care diseases of the World Health Organization (WHO) [38]. Since 2009, the WHO reported that about 80% of the world’s population did not have adequate access to pain relief [39]. Besides, according to the latest data found, 1.5 billion people in the world suffer from chronic pain, and it is estimated that the prevalence of chronic pain fluctuates between 11 and 40% of the population, of which up to 10% report that pain is severe [40,41]. Pain brings with them a series of comorbidities that end up completely affecting the quality of life, and becoming “per se” in a disabling disease. This review is focused on the effect of pomegranate on different nociception models, although the species was being used ancestrally for this purpose in traditional medicine. So the objective is to carry out a document of the existing scientific literature, the interventions performed, comparisons, results, and study designs in which the analgesic effect suggested by traditional medicine was verified (the review includes reports of studies of the pomegranate tree, including all its parts).

There are a wide variety of animal models used in the study of pain. Table 1 describes pain models used in pomegranate studies.

## 2. Results

From a total of one hundred six articles, only 22 (21 preclinical and one clinical) meet the inclusion criteria. Table 2 (later in the document) was constructed from the analysis of data based on the part of the pomegranate studied, the pain model used, the species chosen for the study, the type of pomegranate sample (extracts, commercial products, juice, seeds, etc.) dose, route of administration, reference drug, and most relevant findings in each article. It was found that there is evidence of the antinociceptive effect by pomegranate, demonstrating, to some extent, that its use in traditional medicine has scientific support. Articles in which the effect of extracts of the whole fruit, the peel of the fruit, the juice and seeds (together), the leaves, and the flowers were used. Likewise, there are publications where the effect of ellagic acid is evaluated, which is a majority compound present in the pomegranate [55], and to which some authors attribute the antinociceptive effect [56,57,58], as well as some other polyphenols [59]. According to the data obtained, most of the extracts used are those of polar nature, with methanol and ethanol being the solvents mostly used. This may be because of the nature of the compounds that are likely responsible for the effect (polyphenols and flavonoids are polar). Therefore, we assume that the authors choose solvents because they are able to extract them in a better way. The doses used in the studies ranged from 10 to 3000 mg/kg, the mode was 100 mg/kg, followed by 200 mg/kg, with a statistic mean of 123.8 mg/kg. In general, the analysis of the Inhibition Pain Index (IPI), carried out that 100 mg/kg of doses extract were effective, finding a dose-dependent antinociceptive response in all cases. The highest IPI was found at 3000 mg/kg weight in the writhing test. It should be noted that in most cases the effect of the extracts was comparable with that of their reference drug (without losing sight of the lower doses of the drugs). The most commonly used reference drug was morphine, and the extracts were close to matching the antinociceptive effect of nonsteroidal anti-inflammatory drugs (NSAIDs) at similar doses of the extracts. The parts of pomegranate with less antinociceptive activity were the leaves and flowers; however, more data are needed because only three studies were found. On the other hand, the part with the greatest antinociceptive effect is peel, a fact of great relevance since the peel is considered a waste product, and by having this effect, the product can be given a plus. In this sense, Quachrif et al. [60] studied the effect of the extract of two different varieties of pomegranate peel from Morocco (Amrouz and Sefri), the first variety was used just by herbalists and the second one is a variety of current consumption. The results showed that both varieties have a good antinociceptive effect, being Sefri the one with the greatest effect in the formalin test, Amrouz in writhing and tail immersion test, and in hot plate test, there were no differences. These models confirm the central and peripheral activity of the pomegranate peel; the Amrouz variety had a greater antinociceptive effect in most of the tests being more potent than Sefri. However, composition studies are recommended in which a hypothesis can be generated as to why this difference exists between varieties. This behavior can be related to the content of polyphenols and free organic acids of each variety. In Table 2, articles of the effect of ellagic acid on antinociception were included, since some of the articles suggest that this is the main responsibility for the antinociceptive effect of pomegranate. However, further in the text, we will see that this effect can be a synergy between several compounds present in the pomegranate, which also have been studied in isolation and reported as antinociceptives.

## 3. Discussion

After the whole revision, it must be said that the pomegranate tree and the different parts studied have antinociceptive activity in greater or lesser proportion on different types of pain (nociceptive, inflammatory, neuropathic, acute and chronic pain models). One of the objectives set for this review was to analyze the mechanisms of action of this effect. In this regard, the authors believe that the beneficial effects of pomegranate on pain are due to its phytochemical compounds. Pomegranate contains ellagitannins, gallotanins, flavonoids as anthocyanins, anthocyanidins and catechins, free organic acids (ellagic acid and gallic acid mainly), alkaloids, saponins, terpenoids, coumarins and fatty acids [55,61] among other groups of compounds. Although in most of the articles the authors agree that the phenolic compounds present in the pomegranate, are responsible for their analgesic and antinociceptive effects, the mechanisms of action have not been fully clarified, and few authors ventured to clarify them. In most cases, the authors attribute the antinociceptive effect of pomegranate to three groups of molecules: polyphenols (ellagitannins, free ellagic acid, tannins, gallotannins, and free gallic acid), flavonoids (anthocyanins), and fatty acids (punicic acid). In other cases, it is also assumed that the effect is given by the alkaloids.

### 3.1. Mechanism of Action of the Principal Compounds of Pomegranate

It has been reported that a pomegranate extract was able to inhibit the production of PGE2 and nitric oxide (NO) induced by inflammatory cytokines in vivo. These mechanisms could be mediated by the inhibition of the release of some endogenous nociceptive mediators and would confirm their central activity through supraspinal nociceptive activation [62]. Gainok et al., [63], suggested that antioxidants (flavonoids and polyphenols) optimize the biological actions of naturally occurring NO in vivo. In fact, antioxidants stabilize NO and prolong its cellular concentration, by protecting it against free radicals as a reactive oxygen species (ROS). NO, as a signaling molecule is synthesized from nitric oxide synthase (NOS) that catalyzes the reaction of molecular oxygen with the substrate amino acid L-arginine (L-Arg) to produce NO. NO acts as a modulator in the spinal cord and dorsal root ganglia through nociceptive pathways and mediates neuropathic pain [12,13,14,15,16].

#### 3.1.1. Tannins

**Figure 1:**
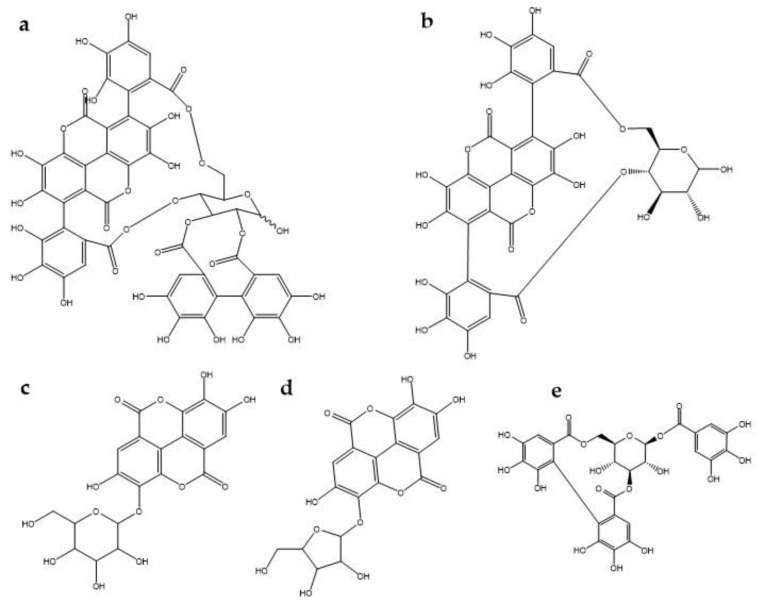
Chemical structure of some tannins involved in antinociception mechanisms. (**a**) punicalagin, (**b**) punicalin, (**c**) ellagic acid hexoside, (**d**) ellagic acid pentoside, (**e**) corilagin.

It has been reported that ellagitannins (polyphenols) are the most abundant bioactive compounds in pomegranate [64]. Pomegranate is a rich source of ellagitannins and ellagic acid derivatives such as punicalagin, punicalin, ellagic acid hexoside, and ellagic acid pentoside and corilagin [65] (Figure 1). Rosillo and Saad [61,66] attribute the antinociceptive effects to tannins. They performed an in-vitro experiment where they exposed aortic endothelial cells to metabolites of ellagitannins (urolithin A glucuronide and its aglycones), which are a consequence of the action of the intestinal microbiota on ellagitannins, to determine the effect on monocyte adhesion and endothelial cell migration, which themselves were significantly inhibited with respect to controls. A decrease in the expression of the inflammatory chemokine CCL2 and Interleukin-8 (IL-8) was also observed [67]. Hollebeeck et al., [68] found that a pomegranate peel extract rich in punicalagin prevented chronic intestinal inflammation in Caco-2 cells. The experiment was performed in an in-vitro model of human intestinal epithelium, acting at the level of gene expression of the proinflammatory molecules interleukin-6 and monocyte chemoattractant protein MCP-1) by direct molecular entrapment and a significant down-regulation of the transcription of these genes was observed. There are also reports of how four free hydrolyzable tannins (punicalagin, punicalin, strictin A and granatin B) showed an inhibitory effect on NO production in RAW 264.7 cells induced by lipopolysaccharide (LPS). Also, granatin B inhibited, to a greater extent, the production of PGE2 and the expression of COX-2 in the same cells. These tannins, especially granatin B, function as an effective anti-inflammatory and have a double effect on anti-inflammation, by decreasing the production of PGE2 in the early stage, and the production of NO in the late stage. [59]. It has been reported that punicalagin- mediated inhibition of PGE2 production in macrophage cells stimulated with LPS was associated with negative regulation of COX-2 proteins [69]. In this regard, Jain et al. [53] observed that a commercial pomegranate fruit extract pretreatment significantly attenuated the increase NO level in a tibial and sural nerve transection induced neuropathic pain model. On the other hand, Karwasra et al. [70] investigated the effect of the pomegranate peel on the nociceptive threshold in acute and chronic cases of inflammation and pain in an animal model of rheumatoid arthritis. They found that 200 mg/kg of pomegranate peel extract showed a significant improvement (p < 0.05) in nociceptive behavior reducing pain, the authors attributed this effect to the presence of high levels of hydrolyzed polyphenols, especially punicalagin, which may be responsible through modulation of NF-κB pathway. In the same way, Moreira et al. [71] showed that corilagin significantly reduced capsaicin-induced nociception, suggesting that this tannin may be involved in the antagonism of the transient receptor potential vanilloid 1 (TRPV1) channel, given that nociception evoked by capsaicin occurs by activation of the TRPV1 channel, which favors release of several chemical (substance P, aspartic and glutamic acids, neurokinin A, calcitonin gene-related peptide, NO) and pro-inflammatory mediators that contribute to increase nociception at central and peripheral levels [72,73].

#### 3.1.2. Flavonoids

**Figure 2:**
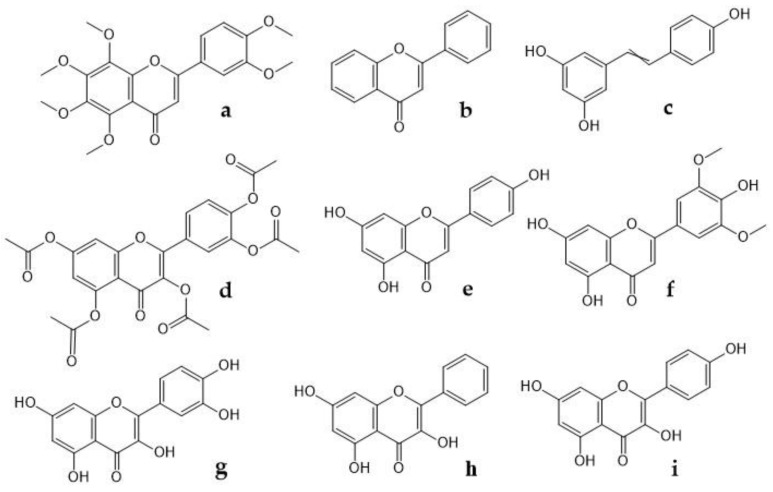
Chemical structure of some flavonoids that modulate COX-2 transcription. (**a**) nobiletin, (**b**) flavone, (**c**) resveratrol, (**d**) quercetin penta-acetate, (**e**) apigenin, (**f**) chrysin, (**g**) quercetin, (**h**) galangin, (**i**) kaempferol.

Flavonoids inhibit COX-2 activity, and it is known that nobiletin, flavone, resveratrol, quercetin penta-acetate, apigenin, chrysin, quercetin, galangin, and kaempferol (Figure 2), modulate COX-2 transcription in Caco2 cells, therefore, they play a role on analgesic activity and its mechanism is to target prostaglandins and inhibiting prostaglandin synthetase and tannins [74]. On the other hand, Takano-Ishikawa et al. [75] studied 39 flavonoids and their related compounds (comparison between subclasses), seeking a relationship between structure and activity of their inhibitory effects on the production of LPS-induced prostaglandin E2 in rat peritoneal macrophages. They found that flavones were the most effective, followed by flavanones and flavonols, which were the least effective. These results suggest that the double C2-C3 bond and the 4-oxo functional group of the C ring are responsible for the high inhibition activities. Likewise, flavonoids showed the greatest inhibitory effect on COX-2 gene expression. Finally, Gao et al. [76] found that Quercetin (20, 40 mg/kg) for 40 and 12 days, respectively in a model of paclitaxel-induced neuropathic pain, suppressed the increased expression levels of TRPV1 in the spinal cords and dorsal root ganglion neurons (DRGs) of paclitaxel-treated rats and mice.

#### 3.1.3. Ellagic Acid

**Figure 3:**
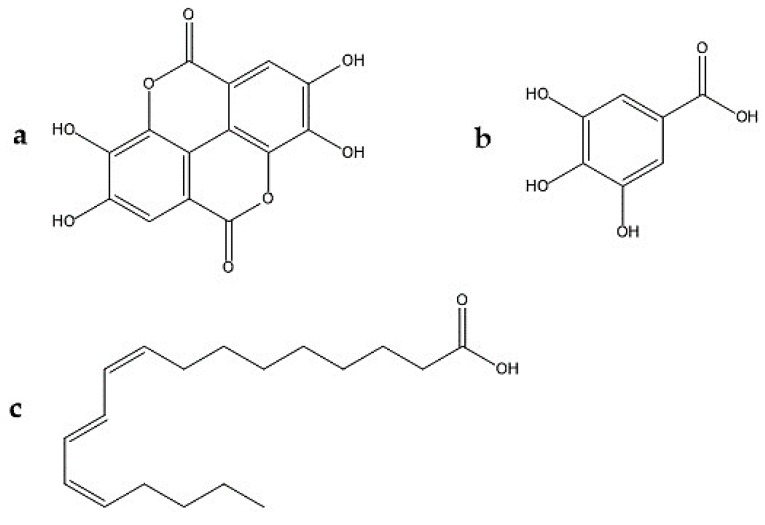
Chemical structures involved in the antinociceptive effect of *Punica granatum* L. (**a**) ellagic acid, (**b**) gallic acid, (**c**) punicic acid.

EA (Figure 3) is an organic acid, derived from gallic acid that occurs in free form, as EA-glycosides or as ellagitannins [77,78]. One hypotheses generated from the mechanism of action of the pomegranate (and its fractions) on nociception is that the EA is one of the responsible. Some studies confirm the antinociceptive activity of ellagic acid in animal models of visceral, inflammatory and cystitis pain. [57,79,80,81,82]. BenSaad et al. [56] suggest that ellagic acid and gallic acid present in the pomegranate extract they evaluated may be the compounds responsible for its analgesic effect. Ghorbanzadeh et al., in 2014, studied whether the L-arginine-NO / cGMP / KATP channel pathway is responsible for the antinociception of EA. It is known that the NO has pro or anti-nociceptive effects, depending on the conditions [83]. Kawabata et al. [58] showed that NO levels are increased in the formalin test by activating NOS at the injection site, which contributes to the induction of nociceptive responses in the second phase of the test. NO can increase the concentration of cGMP, which will lead to the activation of potassium channels. The opening of these channels induces membrane hyperpolarization and reduces depolarization and action potential. Therefore, Ghorbanzadeh et al., showed that prior treatment with L-arginine, NꙌ-nitro-L-arginine methyl (L-NAME) (non-selective NOS inhibitor), sodium nitroprusside, methylene blue and glibenclamide, before the formalin test, were not effective in altering the antinociceptive effect of EA in the first phase of the test. Demonstrating that the antinociceptive effect of EA in this phase was not mediated by activation of the NO / cGMP / KATP channel pathway. On the other hand, it was shown that L-arginine and sodium nitroprusside potentiated the late-stage antinociceptive effect, and it was found that the effect of EA depends on the activation of the L-arginine / NO pathway. As there was a significant dose-dependent reduction in EA-induced peripheral antinociception after intraplantar administration of L-NAME in the late phase of the formalin test. When applied alone, L-NAME (100 μg) did not produce any significant change. Therefore it has no hyperalgesia or anti-hyperalgesia effect, and the effect of EA is partly due to the local generation of NO. In other studies, it was shown that systemic administration of EA produced clear dose-dependent antinociceptive effects in both phases of the formalin test, finding that in the second phase, it was more sensitive to EA. Their results indicated that systemic analgesic effects and peripherals of EA depend on the opioid system, since prior treatment with naloxone, an opioid receptor antagonist, reversed the antinociception caused not only by i.p. EA, but also i.pl. [79,80,81,84]. Also, the analgesic action of EA has been explained by the inhibition of cyclooxygenase, which synthesizes prostaglandins at the sites of peripheral cell damage [85]. 

#### 3.1.4. Gallic Acid

Gallic acid (GA) (Figure 3) is a trihydroxybenzoic acid in which the hydroxy groups are at positions 3, 4, and 5 [86]. BenSaad et al., [87], confirmed in 2017 that gallic acid reduced NO production in RAW2674.7 cells treated with LPS. Gallic acid was also shown to inhibit the production of PGE-2 and effectively decreased to IL-6 in this study. All these effects were dose-dependent. They also found that the expression of the COX-2 gene of RAW264.7 cells induced by LPS was not affected after treatment with gallic acid for 24 h. In addition Santos et al., [88] observed an antihyperalgesic and antinociceptive effect by gallic acid in relation to the dose in nociception animal models. GA significantly inhibited substance P and bradykinin-induced hyperalgesia in the rat leg but did not affect the hyperalgesia caused by prostaglandin E or carrageenan. In addition, GA, in contrast to the reference drug (morphine), was ineffective in the hot plate test in mice. The antinociception produced by GA i.p. in the formalin test was significantly reversed with G. pertussis toxin, i.c.v. and by intrathecal administration of K + channel blockers such as glibenclamide, apamine and caribotoxin, but not by tetraethylammonium. In contrast, GA antinociception was not affected by intraperitoneal treatment with naloxone or with nitric oxide precursor, L-arginine, and this action was not secondary to its anti-inflammatory effect, nor was it associated with nonspecific effects such as muscle relaxation. or sedation, as can be said, that GA produces a pronounced and dose-dependent systemic, spinal, and supraspinal antinociception in mice, perhaps by the activation of K + channels and by a mechanism sensitive to G. pertussis toxin. Finally, in another study, Trevisan et al., [89] considered identifying GA as an antagonist of transient receptor potential ankyrin 1 (TRPA1) channels, this channel play an integral role in pain and neurogenic inflammation via sensory nerve activation at both central and peripheral level (**72,73**) and observing its antinociceptive effects in different pain models in Swiss mice. First, they evaluated the ability of GA to affect the calcium input induced by cinnamaldehyde; then they observed the antinociception by oral administration of GA (3–100 mg/kg) after intraplantar injection of TRPA1 agonists (allyl isothiocyanate, cinnamaldehyde or hydrogen peroxide) in an inflammatory pain model (carrageenan injection i.pl.), and a model of neuropathic pain (chronic constriction injury). GA reduced calcium entry mediated by activation of TRPA1, decreased nociception caused by allyl isothiocyanate, cinnamaldehyde, and hydrogen peroxide. Carrageenan-induced allodynia and edema were greatly reduced by GA treatment. Besides, the administration of GA was also able to decrease cold and mechanical allodynia in the neuropathic pain model. Therefore, GA was found to be a TRPA1 antagonist with antinociceptive properties.

#### 3.1.5. Punicic Acid

Punicic / Punic acid (PUA), also called trichosanic acid (Figure 3), is an omega-5 long chain polyunsaturated fatty acid and an isomer of conjugated α-linolenic acid with structural similarities to conjugated linoleic acid and α-linolenic acid [90]. This fatty acid caused a dose-dependent increase in the peroxisome proliferator activator receptor (PPAR) alpha and gamma indicator activity in 3T3-L1 cells and bound, although weakly, to the ligand binding domain (LBD) of human gamma PPAR. Dietary PUA suppressed NF-kappaβ activation, TNF- α expression and PPAR α and γ response genes regulated upward in skeletal muscle and adipose tissue. The loss of PPAR γ affected the ability of diet PUA to improve glucose homeostasis and suppress inflammation in obese mice. Boussetta et al. [91] demonstrated that PUA exerts a potent anti-inflammatory effect through inhibition of TNF-α-induced priming of NADPH oxidase by attacking p38MAPKinase / Ser345-p47phox-axis and releasing myeloperoxidase in a rat model of 2, 4, 6- trinitrobenzenesulfonic acid (TNBS) induced colitis. 

### 3.2. Toxicity

It is relevant that once the mechanisms of action and the doses used in the different pain models have been studied, we know the toxicity of the studied parts of the plant. The consumption of the edible parts of the pomegranate and its extracts is considered safe. It has shown that a food grade pomegranate fruit extract, administered orally, in an acute toxicity study, had a LD_50_ of more than 5000 mg/kg in Wistar rats and Swiss albino mice and in the sub chronic study all animals survived and no changes in clinical parameters (physical and behavioral) were observed [92]. In other study, the result of the oral acute toxicity (OECD 2001), revealed no signs of toxicity in a peel extract at fixed sole dose of 2000 mg/kg for 14 days [93]. In addition, the butanolic fraction of methanolic extract of pomegranate peel was evaluated in an acute toxicity test, finding that it, was safe at a dose above 500 mg/kg [94]. However, some extracts from other parts may have toxic effects at high doses due to their high content of alkaloids and tannins. Part of the pomegranate tree can also influence the degree of toxicity, for example, it was found that LD_50_ of peel extract (intraperitoneally administration of two varieties from Morocco) is 320.5-355.8 mg/kg in Wistar rats and 300-348.2 mg/kg in albino mice respectively [60]. Vidal et al. [95], found that LD_50_ of hydro alcoholic whole fruit extract of pomegranate administered intraperitoneally to mice was 731.1 mg/kg and found that there are embryotoxicity at a dose greater than 100 mg/kg. It has also been reported that punicalagins (an abundant tannin in pomegranate), has a cytotoxic effect on cell lines, at high concentrations [96]. However, Cerdá et al. [97], conducted a trial in which they concluded that oral administration of high doses (a commercial diet containing 0.5, 2, 5, 10 and 20% of peel extract) of punicalagin from pomegranate elagitannins to Wistar rats for 37 days is not toxic, and corroborated it with an histopathological study of liver and kidney.

## 4. Materials and Methods

### 4.1. Search Criteria

A search for information on the subject was carried out, using as inclusion criteria articles published from 2000 to September, 2019. Only peer reviewed articles published, with no language restrictions were considered in this study (unpublished data were not included). Articles which includes information of pomegranate and pain (nociception) were selected with the keywords: pomegranate, *Punica granatum* L., antinociceptive, analgesic, pain, anti-inflammatory, neuropathic, pomegranate seed, leaf, peel, aerial parts, juice, in repositories such as PubMed, ScienceDirect, Cochrane Library, Worldwide science, Springer link, Refseek, for investigate and review the literature. 

### 4.2. Data Extraction

Data were collected, summarized, analyzed, compared, discussed, and the conclusions were made accordingly. Data extracted from each study included pain model, type of pain, part of pomegranate studied, species studied, and type of extract or compound, dose and route of administration, reference drug, results and an analyses of the mechanisms of action. Studies in which the objective was the exclusive evaluation of the anti-inflammatory effect were not taken into account. The reviewers extracted the data through an analysis of the interventions, the results of their viability were also compared, taking into account the study designs of the articles.

**Table 2:** Effect of different parts of pomegranate on nociception and analgesia in many pain models.

Part of the Pomegranate Tree	Pain Model	Species	Sample	Doses and Route of Administration (mg/kg)	Reference Drug (mg/kg)	Results (*p* < 0.05) and IPI (%)	Ref.
***Whole fruit***	Formalin test	Adult male Swiss albino mice	Commercial extract (high ellagitannins content)	10, 30, 100 i.p.	Diclofenac 100 i.p.	The 100 mg dose reduced nociception in both phases of the formalin test IPI: Not reported	[98]
	Writhing test Hot-tail flick test Plantar test	Adult male albino mice Adult male Sprague Dawley rat	Ethanolic Extract	100, 150, 200 i.p.	Acetylsalicylic acid (ASA) 100 i.p.	Reduce writhes, flicks and hyperalgesia in plantar test IPI: Writhing: 37 (59 ASA) Hot tail flick: 24 (37.5 ASA)	[99]
	Writhing test Hot tail-flick test Plantar test	Adult male albino mice Adult male Sprague Dawley rat	Ethyl acetate extract	100, 150, 200 i.p.	ASA 100 i.p.	Reduce writhes, flicks and hyperalgesia in plantar test IPI: Writhing: 41 (53 ASA) Hot tail flick: 30.5 (43.8 ASA)	[56]
	Writhing test Hot-plate test Tail immersion test	Adult male albino mice	Hydro-alcoholic extract	1000, 2000 and 3000 *per os*.	ASA: 100 *per os*	Reduce writhes Increased time of reaction latency in the tail immersion test in response to thermal stimulation IPI: Writhing: 1 g = 45.3, 2 g = 70.9, 3 g = 86.8	[62]
	Tibial and sural nerve transection	Adult Wistar rats of either sex	Commercial Fruit Extract	100, 300 *per os*	Gabapentin 100 *per os*	At both doses significantly attenuated the biochemical changes (TNF-α, TBARS, GSH and Nitrite) and behavior (hyperalgesia and allodynia) induced by nerve surgery. Also attenuated the tibial functional index. IPI: No data	[53]
***Fruit peel***	Osteoarthritis	Adult women	Hydro-alcoholic extract	500 each 12 hours during 8 weeks *per os*	No data	A decrease in the KOOS (instrumental evaluation of knee injury and score of the result of osteoarthritis), and a decrease in the visual analog scale IPI: No data	[100]
	Hot-plate CFA	Adult Wistar rats (sex not specified)	Lyophilized powder of hydro-alcoholic standardized peel extract	50, 100, 200 *per os*	Indomethacin: 3 *per os*	The resistance to thermal nociception was greater at doses of 200 mg/kg (*p* <0.05), finding an effect comparable to the reference drug. IPI: 58–75% In CFA test, treatment suppressed inflammation of the hind leg and bone damage, and at 200 mg/kg, against changes related to CFA	[70]
	Hot-tail flick test	Male Wistar rats	Hydro-alcoholic extract	100, 200 *per os*	Indomethacin 20 *per os*	100 and 200 mg/kg of hydroalcoholic extract produced analgesic activity comparable to the reference drug. IPI: No data	[50]
	Writhing test Formalin test Hot-plate test Tail immersion test	Adult male Wistar rats Adult albino mice	Methanolic extract	10, 25, 50, 100, 150 i.p. and i.c.v	Morphine sulfate: 5 i.p and i.c.v. DL-lysine acetylsalicylates: 100 i.p. and i.c.v	Produced an antinociceptive effect in all tests. In the hot-plate and immersion test, increase dose- dependent reaction latency to thermal stimuli IPI: Formalin: 75- 82 Writhing: 29- 52	[60]
	Formalin test Writhing test	Adult male Albino mice	Hydroalcoholic extract	400 i.p.	No data	Pomegranate peel extract has analgesic and anti-inflammatory effect in formalin and acetic acid models. IPI: Not reported	[101]
	CFA-induced polyarthritis von Frey test Formalin test	Adult male Wistar rats Adult male ICR mice	Methanolic extract Ellagic acid as marker compound	300 for CFA100 for formalin test Topic formulation	1% diclofenac gel	In CFA and mechanical hyperalgesia test it was found that ellagic acid was only effective at 0.65 and 0.32%, and both (EA and pomegranate peel extract showed significant topical analgesic activities). IPI: Formalin test phase 1 was not inhibit. Formalin test phase 2: 35.63 for extract and 33.76 for EA	[102]
	Painful diabetic neuropathy Hot-plate Tail flick	Alloxan-induced DM adult male Swiss-Webster mice	Spray-dried biopolymeric dispersions from ethanolic extract (commercial) Gallic acid	25, 50 and 100 i.p.	Tramadol: 10 per os Glibenclamide: 5 per os	The extract improved peripheral nerve function in all latency tests, in hot-plate latency compared to control group by 33.3, 73.5, and 85.1% in doses of 25, 50, and 100 mg/kg, respectively.	[52]
	Ethanol (80%) and ASA induction of gastric ulcer	Adult male Wistar rats	Methanolic extract	250 and 500	No data	The extract has a gastroprotective effect IPI: 22.37 and 74.21% Ethanol ulcer 21.95 and 63.41% ASA ulcer	[48]
***Juice and seed***	Hot- tail flick test Hot- plate test	Old and young male Swiss mice	Ethanolic seed extract	100, 250, 500 *per os*	Morphine sulfate 5 *per os*	The seed extract revealed an antinociceptive property (being similar to the reference drug) at doses of 250 and 500 mg/kg. There were no differences due to age of the mice.	[103]
	Writhing test CFA	Adult male Swiss albino mice	Seed oil + ketoprofen nanoemulsion	300 + 10 i.g. Doses response curve: 30- 150 + 0.1- 5 i.g.	No data	The nanoemulsion reduced writhes with an effect of 12 hours maximum compared to ketoprofen (3 hours). Also an effect was found in the dose- response curve at a dose of 1 mg/kg for the nanoemulsion and 0.5 mg/kg for ketoprofen. The evaluation of mechanical allodynia in the CFA test showed that nanoemulsions had an effect up to 10 hours, compared to ketoprofen (6 hours) IPI: Not reported	[104]
	Hypertonic saline- induced corneal pain	Adult male mice (unspecified strain)	Juice and seed extracts	2, 4, 6 mL/kg acute doses *per os* 1, 2, 3 mL/kg chronic *per os*	Morphine: 2 s.c. Naloxone: 2 s.c.	The results showed that the high acute dose, and the lower dose in the chronic test, can decrease acute corneal pain and enhance morphine induced nociception	[105]
***Leaves and flower***	Hot-plate test	Adult male Swiss albino mice	Chloroformic, methanolic and aqueous extract of flower	50 i.p.	Morphine sulfate 5 i.p.	The reaction time after the injection was longer than the control group, and similar to the effect of morphine. The time at which the maximum analgesia was observed for the three extracts was 60 minutes IPI: Not reported	[106]
	Writhing test	Adult male Swiss albino mice Adult male Long Evans rats	Per-ether, dichloromethane and methanol	200 *per os*	Diclofenac: 50 *per os*	Extracts and diclofenac have induced significant decrease in the number of writhes when compared to the control groups. IPI Diclofenac 61.8, Pet-ether 75.7, dichloromethane 68.5, methanol 54.7	[107]
	Writhing test	Either sex adult Swiss albino mice	Hydro alcoholic extract of leaf and peel	100 and 200 *per os*	Ibuprofen 100 *per os*	The leaf and the peel at both doses decreased the number of contractions compared to the control group, the leaf having a lower IPI than peel at both doses. IPI: Leaf: 27.8 and 43.2 Peel: 30.1 and 49.8	[108]
***Ellagic acid as possible responsible for the effect***	Formalin test	Adult male Wistar rats	Ellagic acid	0.03, 0.06, 0.1 0.2 i.p. i.pl.	Morphine: 25 μg/paw	EA induced local and peripheral antinociception in both phases of the formalin test (doses of 100 and 300 µg/Kg)	[57]
	Formalin test	Adult male Wistar rats	Ellagic acid	1, 3, 10, 30 i.pl	Morphine 5 i.p. Indometacin 10 i.p.	The ipsilateral administration of EA into the right paw produced a dose- related antinociception local peripheral during both phases of the test (comparable with morphine) IPI: not reported	[80]
	Writhing test Hot-plate test	Adult male Swiss albino mice	Ellagic acid + morphine	Writhing: EA 1-30 i.p. Morphine 0.25- 3 i.p. Hot-plate: morphine 10 s.c. + EA 1-10 i.p.	No data	EA (1-30 mg/kg) showed significant and dose- dependent antinociceptive effects in the writhing test, interacted with morphine in analgesia in a synergistic manner and also exerted an algic activity on the hot- plate test (with morphine effectively blocked the development of tolerance to morphine analgesia). IPI: Not reported	[79]
	Writhing	Adult male Swiss albino mice	Ellagic acid + Venlafaxine (VLF)	EA 0.3, 1, 3, 10 i.p. VLF 3, 10, 30, 60 i.p.	No data	A dose- dependent inhibition of the contortion response was achieved. The effective dose 50 (ED_50_) versus contortion behaviors were 1.02 (0.86- 1.19) mg/Kg, and 12.37 (9.74- 15.37) mg/ Kg for EA and VLF respectively, also with higher power than VLF, so the combination of EA and VLF has a synergistic interaction.	[81]

Abbreviations: CFA= Complete Freund´s adjuvant, i.p. = intra peritoneal, i.g. = intra gastric, i.c.v. = intracerebroventricular, i.pl. = intraplantar, per os= oral route, s.c.= subcutaneous.

## 5. Conclusions

Through the reviewed articles, it has been proven that pomegranate and its different parts have an antinociceptive effect, through several preclinical pain models and a clinical model, paying particular attention to the effect of the fruit peel. It was described with certainty that certain compounds are responsible for the antinociceptive effect; however, the extracts have hundreds and even thousands of compounds, so there are still a large number of compounds that can be isolated and evaluated (not only polyphenols, flavonoids or fatty acids). The results are conclusive, given the certainty that pomegranate can be considered in the treatment of pain and could have a positive impact on the lives of people with acute and chronic pain. Pomegranate can be used primarily in inflammatory and nociceptive pain, but also neuropathic.

## 6. Prospects

More information is needed about the synergic effect of pomegranate or its compounds identified with drugs in order to test whether there is synergism, additivity, or antagonism of the extracts or compounds of pomegranate and to enhance the effectiveness of existing drugs, with the least number of undesirable effects, by reducing the effective doses thereof. It is also considered necessary that we have the development of more clinical studies where it the effect is proven (if these studies are to be tested in humans, regardless of toxicity reports, not only the bioactivity of the evaluated extracts should be sought, but also the chemistry of the extracts and the biological effect they may have in addition to the antinociception). It is also suggested that we with different extraction methods and solvents (for example, using non polar solvents) for those groups of compounds present in the various parts of the pomegranate and that have not been studied, such as alkaloids, saponins, and terpenoids, this because the antinociceptive effect possessed by the compounds identified in this article, could being enhanced by the presence of other groups of compounds and without knowing it, the effect is attributed only to antioxidant compounds. Finally, it is proposed that we evaluate more mechanisms of action with antagonists that have not been used by the authors cited in this review.

## Figures and Tables

**Table 1:** Pain models used in pomegranate studies.

Model of Pain	Type of Pain	Description	Reference
Formalin test	Nociceptive and inflammatory	Formalin (1-5%) is injected into the dorsal surface of a hind paw (ipsilateral) in rats or mice. The time the animal spent licking, biting, or shaking is measured. Two types of pain are measured at different test periods: from 0 to 15 minutes (phase 1- nociceptive pain), from 15 to 60 minutes (phase 2- inflammatory pain).	[36,42]
Writhing test	Inflammatory (visceral pain)	In the writhing test (mice), the antinociception is evaluated by counting the number of writhes after a parenteral administration of irritating agents (usually 0.6% acetic acid) during three periods of 10 min. A writhe is defined as the abdominal constriction and stretching of at least one hind limb.	[43]
Tail flick test	Nociceptive	The tail-flick test (usually in rat) uses equipment in which a small thermocouple contacts the dorsal surface of the tail near the radiant heat stimulus. A computer system is used to program the temperature and record the latency of the tail in relation to the time taken to move the limb.	[44]
Hot- plate test	Nociceptive	In the classical hot plate test, murines (usually rat) react by licking their paws, jumping or both, and the number of reactions in a period of time is measure The latency time is measured from when the animal is placed on the surface of the hot plate until the moment the animal licks its leg or jumps to avoid thermal pain.	[44]
Tail immersion test	Nociceptive	In the tail immersion test (rat) baseline latency is the time from immersion of the tail in warm water (47 °C) until the appearance of tail-flicking, vocalization or struggling as a nociceptive response.	[45,46]
Corneal pain	Nociceptive	In this model, acute corneal nociception is induced (1 hour after administering the drug to be studied), with a drop of an irritant (usually 5 M NaCl) on the surface of the cornea of rats and the number of eye wipes is measured during 30 s.	[47]
Gastric ulcer	Inflammatory	Gastric ulceration models are carried out with different drugs in acute or chronic treatments (in rats). Usually acetylsalicylic acid (400 mg/kg i.g.), indomethacin (20 -50 mg/kg i.g.) and 80% ethanol. Subsequently, the ulcer index (histopathology) is calculated.	[48,49,50]
Complete Freund’s adjuvant (CFA)	Inflammatory	CFA (a solution of antigen emulsified in mineral oil and inactivated dried mycobacteria) stimulates cell-mediated immunity and leads to potentiation of T helper cells that produces effector T cells and immunoglobulins. It is used exclusively in animal research, due to its painful reaction and potential for tissue damage. It is administered in the paw with a subcutaneous injection of 0.5 ml in rats.	[51]
Diabetic neuropathy	Neuropathic	It is reported that after six weeks of induction of diabetes with alloxan (180 mg/kg i.p.) every 48 h, three times, mice or rats generate diabetic neuropathy, corroborated by tests of hyperalgesia and allodynia, inducing a neuropathic pain model.	[52]
Tibial and sural nerve transection	Neuropathic	The rats are anesthetized, an incision is made in the thigh, and a cut is made directly through the biceps femoris muscle to expose the sciatic nerve and its three terminal branches, two of which are ligated and cut (tibial and sural). The muscle and skin close and over time, hyperalgesia and allodynia are tested to corroborate neuropathy.	[53,54]
